# P-1166. Outcome of Fungemia Caused by Yeast in Children: Significance of Breakthrough Fungemia and Source Identification

**DOI:** 10.1093/ofid/ofae631.1352

**Published:** 2025-01-29

**Authors:** Taketo Kasai, Masaki Yamada, Takanori Funaki, Chikara Ogimi

**Affiliations:** National Center for Child Health and Development, Setagaya, Tokyo, Japan; National Center for Child Health and Development, Setagaya, Tokyo, Japan; National Center for Child Health and Development, Setagaya, Tokyo, Japan; National Center for Child Health and Development, Setagaya, Tokyo, Japan

## Abstract

**Background:**

Fungemia by yeast is a life-threatening infection that occurs in patients with special conditions. It is often identified by its morphology, triggering the empiric therapy. However, the management can be difficult as identification and susceptibility tests require several days, and evidence on management and outcomes in pediatric patients is limited. Thus, this study aimed to characterize pediatric fungemia by yeast and assess their outcomes to help optimize management.

Risk factor analysis for overall mortality following fungemia by yeast

**Figure:**
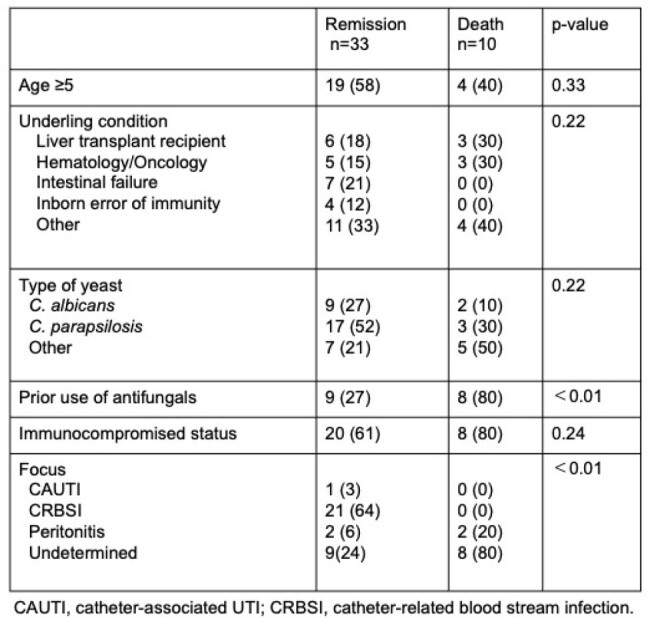

**Methods:**

This is a single-center, retrospective observational study at the largest tertiary children’s hospital in Japan. Electronic medical records of pediatric patients < 18 years who developed fungemia by yeast from April 2014 to March 2024 were reviewed. To simplify, the data on the first fungemia by yeast was collected on patients with multiple episodes, along with patient characteristics, details of identified organisms, use of antifungals, and prognosis. Breakthrough fungemia was defined when it occurred during the use of systemic antifungals for ≥ 3 days. Descriptive data, the risk for overall mortality and survival following fungemia were analyzed using chi-square test and Kaplan-Meier curve analysis with a p-value < 0.05 being considered significant.

Overall survival following fungemia by yeast according to prior use of antifungals

**Figure:**
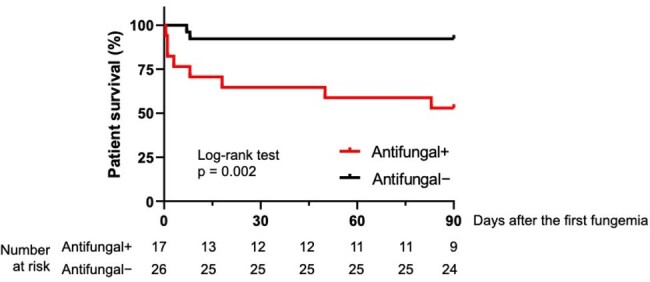

**Results:**

Overall, 55 episodes of fungemia by yeast were identified in 43 patients. 28/43 (65%) patients were immunocompromised. *Candida parapsilosis* was the most frequently detected (20/43, 47%), followed by *C. albicans* (11/43, 26%). Breakthrough fungemia occurred in 17/43 (40%) cases. Mortality was significantly higher in patients with breakthrough fungemia compared to those without prior antifungals (8/17 (47%) vs. 2/26 (8%), p< 0.01). In addition, when the source of fungemia was clinically undetermined, the overall mortality rate was high (8/17, 47%, p< 0.01) (Table). Further, the survival curve after the onset of fungemia by yeast depicted the worse outcomes in patients with breakthrough fungemia (p< 0.01) (Figure).

**Conclusion:**

In pediatric patients with fungemia by yeast, the mortality rate was higher in breakthrough fungemia and fungemia with unknown sources. Therefore, it is crucial to recognize the importance of breakthrough fungemia and identifying the source of infection to pursue appropriate source control.

**Disclosures:**

**Chikara Ogimi, MD, PhD**, AstraZeneca: Honoraria|bioMerieux Japan Ltd.: Honoraria|ELSEVIER: Honoraria|KYORIN: Honoraria|Miyarisan: Honoraria|MSD: Honoraria|NOVARTIS: Honoraria|Pfizer: Honoraria

